# Dataset for the performance of 15 lumbar movement control tests in nonspecific chronic low back pain

**DOI:** 10.1016/j.dib.2022.108063

**Published:** 2022-03-16

**Authors:** Elisabeth Adelt, Thomas Schöttker-Königer, Kerstin Luedtke, Toby Hall, Axel Schäfer

**Affiliations:** a University of Applied Sciences and Arts Hildesheim, Faculty of Social Work and Health, Germany; b University of Luebeck, Institute of Health Sciences, Department of Physiotherapy, Pain and Exercise Research (P.E.R.L.), Luebeck, Germany; c Curtin School of Allied Health, Curtin University, Perth, WA, Australia

**Keywords:** Movement control, Item response theory, Structural validity

## Abstract

The ability to actively control movements of the lumbar spine (LMC) is believed to play an important role in non-specific chronic low back pain (NSCLBP). However, because NSCLBP is a multifactorial problem and LMC a complex ability, different aspects of LMC are still debated including the influence of pain, the question whether LMC is a cause or consequence of NSCLBP or whether differences in LMC are due to population variance. The complexity of LMC is reflected in the large number of described tests, hence it is not possible to evaluate LMC by a single test. LMC ability should be understood as a latent construct. The structure of LMC and how to summarize results of different single LMC tests is unknown. The dataset provided in this article was used to analyse the structural validity of LMC in NSCLBP. 277 participants (age 42.4 years (± 15.8), 61% female) performed 15 different test movements. 21 experienced physiotherapists rated the performance of each test movement on a nominal scale (correct/incorrect including the direction of test movement). A test was rated as “incorrect” if movement in the lumbar spine occurred prematurely and/or excessively based on the visual observation of a trained physiotherapist. In addition to the judgement whether the test performance was correct/incorrect the direction of test movement and the presence of pain was noted. For statistical analysis, raw data was converted to a binary scale (correct/incorrect). Item response theory (IRT) is recommended to analyse the data because the underlying statistical model is reflective, the single LMC tests are binary scaled (correct/incorrect) and the underlying ability (LMC) measured on a continuous scale. First dimensionality and local independence were analysed, followed by selection of the best fitting IRT model. Finally, IRT modelling was used to describe the psychometric properties of each item and each battery of tests. The datasets provided in this article are useful for calibration and for group comparisons. Besides they support a better understanding of LMC. ***Link to publication of original article in “musculoskeletal science and practice”***

**Specifications Table**

**Table:** 

Subject	Physical Therapy and Rehabilitation
Specific subject area	Movement control Lumbar movement control tests Physical therapy Non-specific chronic low back pain
Type of data	Table Figure
How data were acquired	Data were collected based on the subjective judgement of a trained investigator. Decision on an incorrect test result was based on eyeballed estimation. All test results were documented using a standardized test protocol (see Appendix 2).
Data format	Raw and Analyzed (binary data without direction and binary data with direction) Microsoft Excel datasheet
Parameters for data collection	Participants were recruited from 19 outpatient physiotherapy clinics in Germany and Austria between April and September 2019. They met the following inclusion criteria: age ≥18 years; ability to understand instructions; NSLBP with or without radiating leg pain; symptoms ≥3 month. Subjects were excluded if they had specific spinal pathologies. All 21 examiners (raters) were physiotherapists (mean age 39.5 years (SD=10.4), 12 males, with a mean of 15.5 (SD=9.8) years of experience). They were trained towards or had attained recognized manual therapy qualification. All physiotherapist were trained in the procedures (test movements and test ratings) for one and a half hours, and provided with additional web-based material. The test performance was rated on a nominal scale (correct/incorrect plus direction of test movement).
Description of data collection	The participants performed 15 active test movements in five different starting positions (standing, sitting, supine, prone and side lying). Participants were evaluated with these tests in individual treatment rooms, performing all LMC tests in one session. The order of testing and instruction were standardized. Each test could be repeated (if failed) up to three times. All test results were documented using a standardized test protocol (see Appendix 2). A test was rated as “incorrect” if movement of the lumbar
	spine occurred prematurely and/or excessively based on the subjective judgement of a trained rater. If the test performance was rated to be incorrect the direction of observed incorrect test movement was also noted.
Data source location	University of Applied Sciences and Arts Hildesheim Faculty of Social Work and Health Hildesheim, Germany
Data accessibility	With the article
Related research article	E. Adelt, T. Schöttker-Königer, K. Luedtke, T. Hall, A. Schäfer. 2021. Musculoskeletal Science and Practice. In Press. DOI: 10.1016/j.msksp.2021.102406

## Value of the Data

• The dataset may help to better understand the complexity of LMC ability.
• The dataset can be processed to investigate the structure of LMC and how single test results can be combined.
• The dataset can be used to compare the ability of LMC in participants with NSCLBP with other groups. The data of other groups or subgroups can be calibrated using the data provided with this article.
• The dataset will be beneficial for researchers and practitioners evaluating and measuring LMC.

## 1 Data Description

The data reported in this article is related to the performance of 15 active test movements by 277 participants with NSCLBP.

Appendix 1 describes each test movement showing photographs with the initial and end position. In addition, the test instructions and the explanation of incorrect test performances are given.

Appendix 2 shows the standardized test protocol used by the raters. It also displays the value (0, 1, 2, 3 or 5 and optional 4) given for each nominal rating.

The raw dataset, presented in Microsoft Excel, is given by three spreadsheets (1 – 3):

The first displays the nominal ratings explained in the test protocol. For the second and third spreadsheet, the data were converted to binary ratings (correct (1)/incorrect (0)). The second spreadsheet displays the ratings without giving attention to the direction of incorrect movement. Following the ratings represented data which did not consider the specific direction (e.g. extension, flexion, rotation/lateral flexion) of incorrect LMC. (non-directional specific). The third spreadsheet displays the ratings where measurements represented the specific direction (e.g. extension, flexion, rotation/lateral flexion) of incorrect LMC (directional specific).

An item number is given to each test rating (item 1 to item 15). For direction-specific ratings a letter indicating the direction of incorrect movement (e = extension, f = flexion, r = rotation/lateral flexion) was added.

Considering the data reported in the Microsoft Excel file Table 1, Table 2, Table 3 presents the statistical analysis of dimensionality.

**Table 1:** Unidimensional LMC model: Explanatory Factor Solution based on tetrachoric correlation matrix for binary, non-direction specific, scored items.

Factors	Factor 1	Factor 2	Factor 3
Eigenvalue	2.79	2.53	2.44
% of variance	18.6	16.9	16.3
Cronbach's alpha	0.57	0.49	0.57

Factor Loadings			

Item 1 (Forward bend)		0.75	
Item 2 (Backward arching)	0.56		
Item 3 (Arm lift)	0.48		
Item 4 (One leg stance)			0.59
Item 5 (Sitting knee extension)		0.48	0.59
Item 6 (Chest drop)		0.72	
Item 7 (Single heel slide)	0.63		
Item 8 (Leg lift & hold)	0.72		
Item 9 (Bent knee fall out)			0.4
Item 10 (Prone knee flexion)			0.61
Item 11 (Single hip extension)	0.72		
Item 12 (Single hip rotation)			0.79
Item 13 (Rocking backward)		0.73	
Item 14 (Rocking forward)	0.66		
Item 15 (Top leg turn)			0.63

Factor Rotation Matrix			

	Factor 1	Factor 2	Factor 3
Factor 1	0.6026	0.5378	0.5896
Factor 2	0.7559	-0.6216	-0.2055
Factor 3	-0.256	-0.5695	0.7811

*Note:* PCA_tetra_ fixed for 3 factors. Rotation method: orthogonal Varimax with Kaiser's normalization. Items with loadings <0.4 were suppressed from the table for ease of interpretation.

**Table 2:** Explanatory Factor Solution based on tetrachoric correlation matrix for binary, direction-specific, scored items.

	Extension Control	Flexion Control	Rotation/Lateral Flexion Control
Eigenvalue of factor 1	1.94	2.09	2.39
explained % of variance	48.5	52.3	47.8
Cronbach´s alpha	0.50	0.46	0.58

Factor Loadings			

Item 2_e (backward arching)	0.66		
Item 8_e (leg lift & hold)	0.74		
Item 11_e (prone single hip extension)	0.65		
Item 14_e (rocking forward)	0.73		
Item 1_f (forward bend)		0.83	
Item 5_f (sitting knee extension)		0.61	
Item 6_f (sitting chest drop)		0.66	
Item 13_f (rocking backward)		0.78	
Item 5_r (sitting knee extension)			0.63
Item 9_r (bent knee fall out)			0.63
Item 11_r (prone single hip extension)			0.52
Item 12_r (prone single hip rotation)			0.83
Item 15_r (side lying top leg turn out)			0.8

Correlation Matrix			
	Extension control	Flexion control	Rotation/Lateral flexion control

Extension control	1		
Flexion control	0.08 #	1	
Rotation/lateral flexion control	0.06 #	0.25 ***	1

e = incorrect LMC in extenion; f = incorrect LMC in flexion; r = incorrect LMC in rotation/lateral flexion

*Note:* Factor analysis Rotation method: orthogonal Varimax with Kaiser's normalization. Items with loadings of less than 0.5 were suppressed from the table for ease of interpretation. Correlation matrix of the estimated Theta`s based on Pearson correlations. #=not significant, ***p <0.001

**Table 3:** Item results included in second statistical model.

				Incorrect (%)				

Direction	Item No.	Test Movement	Miss. Values (%)	+ P^1^	Total^2^	First Step Removed	Second Step Removed	Finally Selected
Extension	2_e	Backward arching	0.4	18.8	41.4			x
	3_e	Arm lift	0	1.8	12.3		x	
	4_e	One-leg-stance	0.4	0	2.9	x		
	7_e	Single heel slide	0	0.4	4.7	x		
	8_e	Leg lift & hold	0	6.5	45.1			x
	10_e	Prone knee flexion	0.7	0.4	16.4		x	
	11_e	Single hip extension	0	6.9	35			x
	14_e	Rocking forward	0.7	3.6	43.3			x

Flexion	1_f	Forward bend	0	3.6	11.6			x
	4_f	One-leg-stance	0.4	0	5.4		x	
	5_f	Sitting knee extension	0	1.4	17			x
	6_f	Chest drop	0.4	1.8	12.7			x
	7_f	Single heel slide	0		1.8	x		
	8_f	Leg lift & hold	0	0.7	2.9	x		
	13_f	Rocking backward	0.7	2.5	21.1			x

Rotation/Lateral flexion	4_r	One-leg-stance	0.4	4.3	39.1		x	
	5_r	Sitting knee extension	0	1.1	24.9			x
	7_r	Single heel slide	0	1.8	36.5		x	
	9_r	Bend knee fall out	0	1.8	48.4			x
	10_r	Prone knee flexion	0.7	0.4	19.3		x	
	11_r	Single hip extension	0	5.4	39			x
	12_r	Single hip rotation	1.1	6.6	46			x
	15_r	Top leg turn out	1.5	5.1	45.4			x

Note: n=277

1 : % of incorrect test performances and pain

2 : total % of incorrect test performances; First step: prevalence <5%; second step: removed based on inter-item correlation and PCA_tetra_

Table 1 reflects the raw data of the non direction-specific scored items 1 to 15. (see Excel spreadsheet 2). The assessment was based on the Kaiser Criterion, which stated that all factors with an eigenvalue >1 are considered as acceptable [1].

Based on inter-item tetrachoric correlation principal component analysis (PCA_tetra_) fixed for 3 factors and Kaiser's normalization, the 3 factors explain 51.8% of the variance. Factor loadings range from 0.4 to 0.79 and are distributed over the three factors, e.g. item 2, 3, 7, 8, 11 and 14 are loading on factor 1 whereas item 8 and item 11 show the highest factor loading (0.72). Factor 2 is reflecting by item 1, 5, 6, and 13. Item 4, 5, 9, 10, 12 and item 15 are loading on factor 3. Items with value <0.4 were suppressed from the table. The Cronbach's alpha for non-direction-specific scored items range from 0.49 to 0.57.

In Table 2 the analysis of the direction-specific data (see Excel spreadsheet 3) is presented. After item selection (see Table 4), the remaining extension-specific, flexion-specific and rotation-specific items were included in the PCA_tetra_. Item 2_e, 8_e, 11_e and 14_e showed unidimensional structure with only 1 factor (eigenvalue 1.9) explaining 48.5% of the variance. For all items, the factor loading was >0.6. The mean item-item correlation of the final four items was 0.31 (range 0.24 - 0.49).

**Table 4:** Comparison of unidimensional vs multidimensional IRT-model using GSEM.

Model	Df	Loglikelihood	AIC	BIC	LRT
UIRT	26	-2043.81	4139.62	4233.85	p=0.0224
MIRT	26	-2022.62	4097.24	4191.46	

N=277, number of items=13 (direction-specific; binary scored); UIRT=unidimensional item response theory model; MIRT=multidimensional item response theory model; Df=number of degrees of freedom; AIC=Akaike information criterion; BIC=Bayesian information criterion; LRT=likelihood ratio tests.

The remaining flexion-specific items (1_f,5_f,6_f and 13_f) showed an eigenvalue of the first factor of 2, explaining 52% of the variance. The loading of all items was between 0.60 (item 5) and 0.83 (item 1), the mean item-item correlation was 0.36 (range 0.2 - 0.54).

The remaining rotation-specific items (5_r,9_r,11_r,12_r and 15_r) showed an acceptable factor structure with one main factor that showed an eigenvalue of 2.4, explaining 48% of the variance. The loading of these items was between 0.52 (item 11_r) and 0.83 (item 12_r), the mean inter-item correlation was 0.33 (range 0.19 - 0.6).

All flexion control items and rotation/lateral flexion control items showed significant correlation (p=0.25).

In Table 3 the item selection of the direction-specific items can be seen. The first step during item selection aimed to remove all items with prevalence <5%. The second step included the removal of items with weak inter-item correlation (correlation <0.2) and weak factor loading (<0.5) based on PCA_tetra_.

After the first step, items 4_e, 7_e, 7_f, and 8_f were removed, because in the relevant direction less than 13 out of 277 item results were incorrect.

For extension control tetrachoric correlation between 2_e and 3_e (r = 0.085), 3_e and 10_e (r = 0.02) and between 8_e and 10_e (r=0.11) were below the desired value of 0.2. Including the remaining items (2_e, 3_e, 8_e, 10_e, 11_e and 14_e) in PCA_tetra_, there were 2 factors with an eigenvalue >1. After the second step, item 3_e and 10_e were removed regarding their low correlations and factor loadings on first factor <0.4. The finally selected items for extension control were item 2_e, 8_e, 11_e and 14_e.

For flexion control, the correlations between item 4_f and 1_f (r = 0.18) and 4_f and 6_f (r = 0.012) were below 0.2. In addition, item 4_f showed 5.4 % of incorrect item results which is just above the defined threshold of 5%. Consequently item 4_f was removed from the further analysis. The finally selected items for flexion control were item 1_f, 5_f, 6_f and 13_f.

For the analysis of rotation/lateral flexion control, tetrachoric correlation showed weak inter-item correlations for item 4_r, 7_r and 10_r (correlations <0.2: between item 4_r/7_r; 4_r/9_r; 4_r/15_r; 7_r/12_r; 9_r/10_r; 10_r/11_r). PCA_tetra_ showed 3 factors with an eigenvalue >1. Item 4_r, 7_r and 10_r were removed because of factor loadings <0.5, loadings >0.5 on a second factor and low inter-item correlation. The finally selected items for rotation/lateral flexion control were item 5_r, 9_r, 11_r, 12_r, 15_r.

Based on the remaining 13 direction-specific items (see Table 4), a unidimensional (UIRT) versus a multidimensional MIRT-model with 3 dimensions using generalized structural equation modelling (GSEM) were compared. The data of both models were presented in Table 4. The Akaike information criterion (AIC) showed lower value (4097.24) for the MIRT as well as the Bayesian information criterion (BIC) which showed a value of 4191.46 for MIRT. The difference in BIC was >10, the likelihood ratio test (LRT) was significant (p = 0.0224).

Table 5 represents the IRT-model selection for each LMC direction (extension, flexion, rotation/lateral flexion). For LMC in extension and flexion the AIC and BIC were lower for one-parameter logistic model (1-PL) than for two-parameter logistic model (2-PL). The LRT was nonsignificant (p=0.60 for extension and for flexion p = 0.16). For LMC in rotation/lateral flexion the AIC was lower for 2-PL and BIC was lower for 1-PL. The LRT was significant (p = 0.002).

**Table 5:** IRT model selection.

LMC in	Modell	Df	Loglikelihood	AIC	BIC	LRT
ext	1-PL	5	-717.86	1445.73	1463.85	p=0.603
	2-PL	8	-716.94	1449.88	1478.87	

flex	1-PL	5	-453.22	916.44	934.56	p=0.163
	2-PL	8	-450.66	917.32	946.31	

rot/lat flex	1-PL	6	-863.14	1738.28	1760.02	p=0.002*
	2-PL	10	-855.02	1730.04	1766.28	

n=277, number of items=13 (binary scored), Df=Degrees of freedom; AIC=Akaike information criterion; BIC=Bayesian information criterion; LRT=likelihood ratio test.

**Fig. 1:**
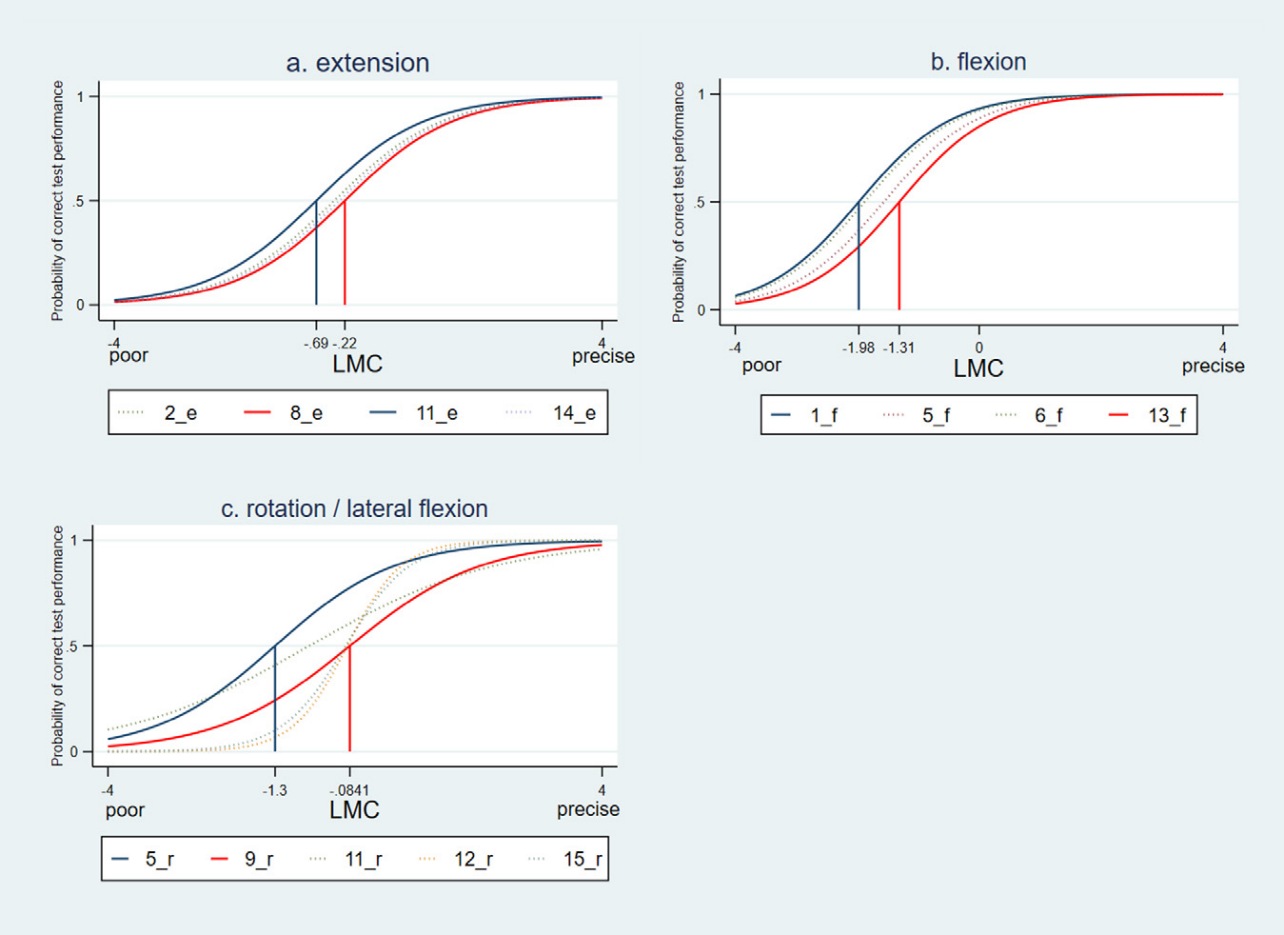
Item characteristic curves (ICC) of a. LMC in extension . b. LMC in flexion, c. LMC in rotation/lateral flexion. The item difficulty values for the easiest (blue solid line) and the hardest (red solid line) were given. Y-axis: probability of correct test performance; x_axis: ability of LMC measured on a Theta scale.

**Fig. 2:**
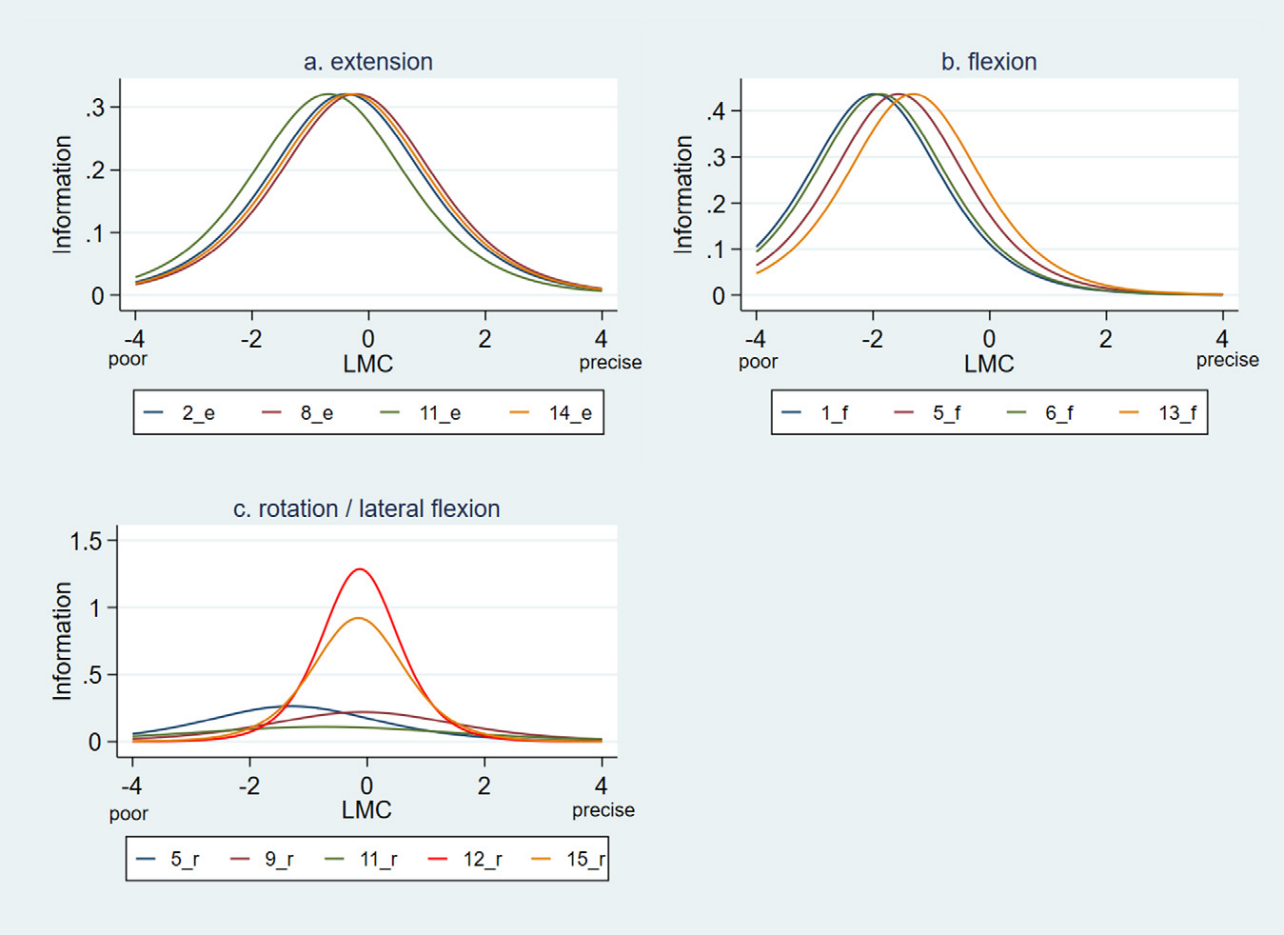
Item information function curves of a. lumbar extension tests. b. lumbar flexion tests, c. lumbar rotation tests. Tall and narrow curves are indicating high information /precision on a narrow ability range of LMC whereas short and wide curves are describing low information/precision on a broad range. For the 1-PL model, used for flexion and extension, all item information parameters are equal for the 2-PL model, used for rotation/ lateral flexion, the value divers according to the discrimination ability.The location of the centre of the curves reflects the difficulty of the items, the height reflects the item discrimination.

**Fig. 3:**
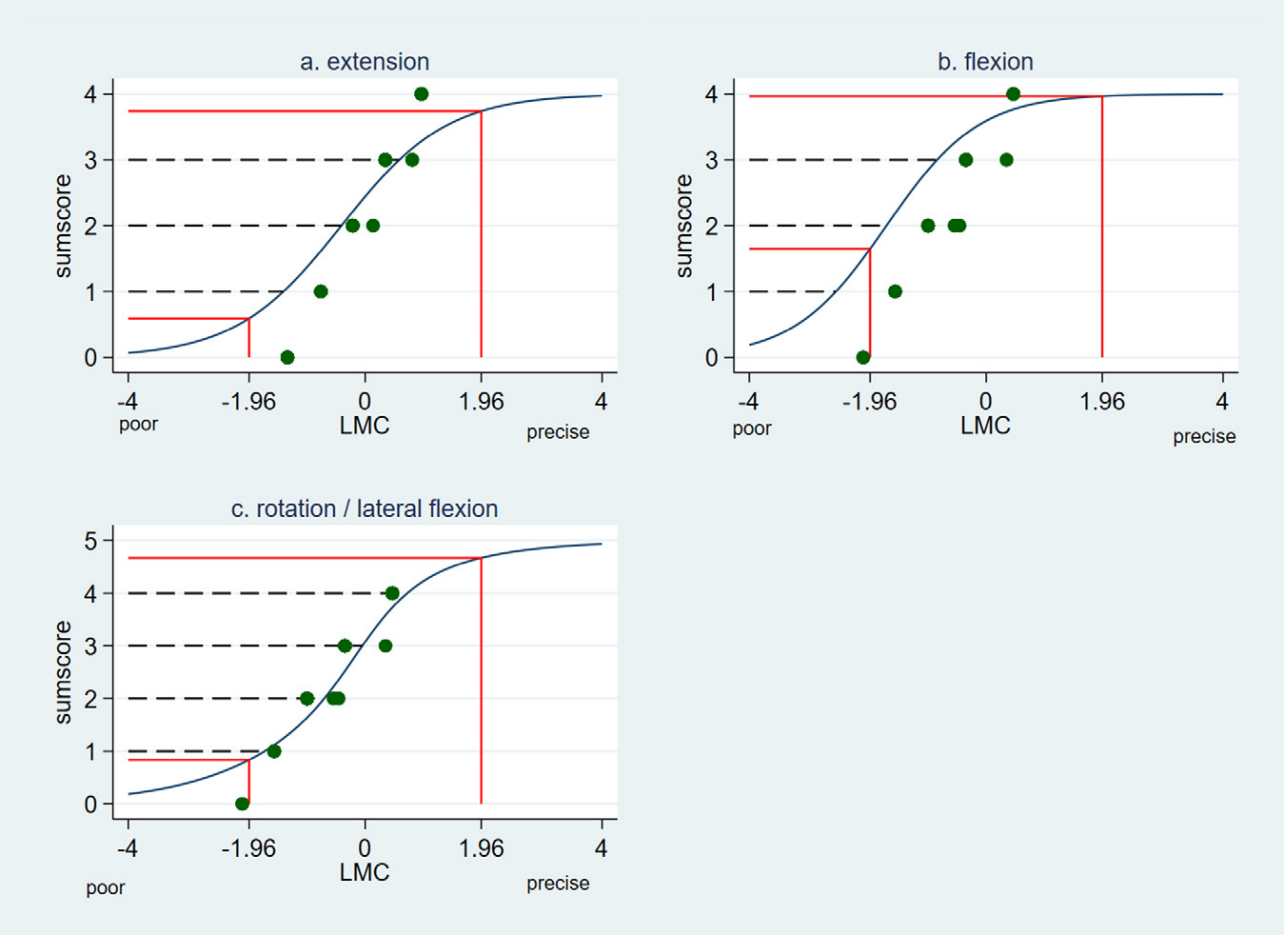
(modified from [3]) Test characteristic curve (TCC) of a. lumbar extension tests. b. lumbar flexion tests. c. lumbar rotation tests. The dotted lines lines visualize the relationship between a given theta (x-axis) and number of correctls performed tests (y-axis).The red solid lines displays the 95% critical values from the normal standard distribution. In addition scatter plots (green dots) between the expected sumscore and the estimated Theta values is shown.

Based on the above selected items, Item response theory was used to create Fig. 1, Fig. 2 and 3. The 1-PL model was used for LMC in flexion and extension and the 2-PL model for estimation of LMC in rotation/lateral flexion:

Fig. 1 displays the estimated item difficulty and discrimination parameters for all selected items. Steeper curves are indicating better item discrimination properties. For the 1PL model, used for flexion and extension, all item discrimination parameters are equal and set to 1.0. For LMC in rotation/lateral flexion Item 12 shows the highest discrimination (see also Fig. 2). The estimated difficulty parameter values for the easiest and the hardest item were given. Items with a negative value are relatively easy, while items with appositive value are relatively hard. The easiest item is placed on the left (blue solid line) and the hardest on the right (red solid line) side. It can be seen that all items for LMC in flexion (range -1.96 to -1.31) are easier than those for extension control (range -0.69 to -0.22). The items for evaluation of LMC in rotation/lateral flexion are covering the highest ability range (-1.3 to -0.08).

Fig. 2 displays the Item information function (IIF) for all selected items. In IRT the item information function replaces item reliability as used in the classical test theory. In IRT the term “information” is used to describe the precision/reliability of an item or a whole test [2]. The standard error of measurement (SEM) is the reciprocal of information, so that more information means less error. Tall and narrow IIFs are indicating high precision on a narrow ability range whereas short and wide IIFs are describing low precision on a broad range Fig. 2. shows high information and therefore high precision for individuals with NSCLBP who's LMC in flexion was 1.9 SD poorer than the average. Whereas extension and rotation/lateral flexion specific items were most informative/precise for participants whose LMC was slightly worse than the average. For LMC in rotation/lateral flexion the test performance to item 12 and item 15 are given the highest amount of information about the ability level of the participant (compare to high discrimination displayed in Fig. 1).

In Fig. 3 the non-linear relationship between the classical sum score and the ability of LMC is presented. The test characteristic curve (TCC) for extension showed correct LMC in 2 out of 4 items for a participant with average LMC ability. Participants with NSCLBP whose extension control is 1.38 SD below average, will have a correct test result in 1 out of 4 items (25%). For flexion, a participant with an average lumbar flexion control will show up to 3 out of 4 correct test results. For rotation/lateral flexion, a participant with LMC control in rotation/lateral flexion that is 1.7 SD poorer than average, will succeed in performing 1 out of 5 items. Participants with an average lumbar movement rotation control (0.04 SD poorer) will have 3 out of 5 correct test results. Using the 95% critical values from the standard normal distribution (-1.96 to 1.96) Fig. 3 displays that it can be expected that 95% of randomly selected people with NSCLBP will score between 1 and 4 when evaluating LMC in rotation/lateral flexion.

In addition a scatter plot was added to the TCCs in Fig. 3. The green spots visualize the relationship between the summated scores versus the predicted ability level of LMC. For example, the ability of LMC in rotation/lateral flexion corresponding to a summated score of 3 ranges across the ability continuum from about -0.5 to 0.5. The clear ordinal structure of the classical summated scores is shown.

## 2 Experimental Design, Materials and Methods

### 2.1 Participants

The characteristics of the participants (n = 277) were given in the related article [3].

The inclusion criteria were age ≥18 years, ability to understand instructions, NSLBP with or without radiating leg pain, symptoms ≥3 month. Subjects were excluded if they had specific spinal pathologies (e.g. fractures, radiculopathy and numbness) [4]. The data were collected in 19 outpatient physiotherapy clinics in Germany and Austria from 21 trained physiotherapists.

### 2.2 Data collection

Participants were recruited from 19 outpatient physiotherapy clinics in Germany and Austria between April and September 2019. They met the following inclusion criteria: age ≥18 years; ability to understand instructions; NSLBP with or without radiating leg pain; symptoms ≥3 month. Subjects were excluded if they had specific spinal pathologies.

All 21 examiners (raters) were physiotherapists (mean age 39.5 years (SD=10.4), 12 males, with a mean of 15.5 (SD=9.8) years of experience). They were trained towards or had attained recognized manual therapy qualification. All physiotherapist were trained in the procedures (test movements and test ratings) for one and a half hours, and provided with additional web-based material.

Based on a literature search, four conceptual frameworks evaluating LMC were identified [5], [6], [7], [8]. 15 tests with at least good reliability (κ≥0.61) and not requiring equipment were selected [9] (Appendix 1). Participants were evaluated with 15 active test movements in five different starting positions (standing, sitting, supine, prone and side lying). All tests were carried out in individual treatment rooms. Participants performed all LMC tests in one session. The order of testing and instructions were standardized. Each test could be repeated (if failed) up to three times. All tests did not require equipment.

An incorrect LMC test was characterized by the inability to control movements of the lumbar spine. A test was rated as “incorrect” if movement in the lumbar spine occurred prematurely and/or excessively based on the subjective judgement of a trained investigator [10]. Decision on a incorrect test result was based on eyeballed estimation. All test results were documented using a standardized test protocol (Appendix 2).

For the direction-specific viewpoint it was nessecary that the incorrect movement was observed into a the specific direction (extension, flexion or rotation/lateral flexion).

## 2.3 Data Processing

The examiners collected nominal data from 15 different movement control tests (correct/incorrect plus direction of test movement: extension, flexion, rotation/lateral flexion) based on eyeballed estimation.

All nominal data were converted to binary data. Variables which distinguish between not directions-specific and direction-specific were created.

The direction of test movement performed on both sides (left and right) was rated to be correct only if they were “correct” on both sides. The direction of test movement which was evaluate incorrect in more than one direction were rated based on the following rule:

• if an incorrect test performance was observed on only one side, rotation/lateral flexion was assumed to be the direction of interest (asymmetric).
• if an incorrect test performance was observed on both sides, flexion or extension was assumed to be the direction of interest (symmetric).

## 2.4 Statistical Analysis

Descriptive statistics were used for demographic and clinical characteristics of the sample.

To evaluate whether LMC should be measured direction-specifically, the underlying assumptions of the statistical IRT model, dimensionality, local independence (LI) and monotony [2], were investigated. Subsequently, based on model and data-fit statistics, the best IRT-model was selected [2]. This analysis included multiple steps:

1. First the hypothesis of dimensionality was investigated. Therefore, two statistical models were compared using inter-item tetrachoric correlations, factor analysis (principal component analysis based on tetrachoric correlation matrix (PCA_tetra_)) and generalized structural equation modeling (GSEM). For the first model the results of all 15 single LMC tests movements were rated to be incorrect or correct without giving attention to the direction of LMC. In this model 15 single item results were included. For the second statistical model, all 15 single LMC test movements were rated to be incorrect or correct according to the 3 possible directions of LMC (lumbar extension, lumbar flexion, lumbar rotation/lateral flexion). Because several test movements could be rated to be incorrect in different directions, e.g. item 5 (sitting knee extension) could be incorrect in flexion or rotation/lateral flexion, these second model included 23 binary scored test results. Tests with a small number of incorrect results (<5%) were removed due to potential ceiling effects.
2. The second step included the selection of the appropriate IRT model. For the final IRT models the assumptions of local independence (LI) and unidimensionality were tested. As proposed by Raykov and Marcoulides [2], the Akaike information criterion (AIC), the Bayesian information criterion (BIC) and the likelihood ratio test (LRT) was used to select the best fitting IRT model for each dimension. The Akaike information criterion (AIC) and the Bayesian information criterion (BIC) were used to compare nested and non-nested models [11]. Lower values are indicative for better model data fit. For interpretation of the differences in AIC/BIC, Raftery [12] suggested the following benchmark: weak (0-2), positive (2-6), strong (6-10) and very strong (>10) evidence for the model with the lower value. In addition the likelihood ratio test (LRT) was apply to compare nested models [2]. The assumption of LI was investigated using Yen's Q3 statistics and unidimensionality was further evaluated applying the Dimensionality Evaluation to Enumerate Contributing Traits (DETECT) [13]. Any pair of items with residuals correlating >0.2 may violate LI [14]. Unidimensionality of the dataset is reached if the DETECT-Index is <0.2 [13].
3. Finally, the selected IRT models were fitted and the psychometric properties including item characteristics, test characteristics and test reliability were calculated. For binary scored items, a 1-PL (Rasch model) or 2-PL (Birnbaum model) logistic IRT-model can be used. The 2-PL model estimates item difficulty (parameter b) and item discrimination (parameter a) for each item. The 1-PL IRT-model is a restricted 2-PL model because it assumes the same discrimination parameter for each item. Therefore the 1-PL model is nested in the 2-PL model. As proposed by Raykov and Marcoulides [2], the AIC, the BIC and the LRT were used for selection of the best fitting IRT-model for each dimension.

## Ethics Statement

The acquisition of this data was approved by the Human Research Ethics Committee of the University of Applied Sciences and Arts in Hildesheim (HAWK), Germany (Approval date 15/02/2019) and followed the recommendations of the Declaration of Helsinki. All participants received detailed information regarding the different procedures and provided informed written consent.

## Supplementary Materials

Supplementary Materials associated with this article can be found as Appendix 1: description of test movements and Appendix 2: test protocol

## Declaration of Competing Interest

The authors declare that they have no known competing financial interests or personal relationships that could have appeared to influence the work reported in this paper.

## Data Availability

Data Availability: Dataset for the performance of 15 lumbar movement control tests in non-specific chronic low back pain (Original data) (Mendeley Data).

## Appendix Supplementary materials

Image, application 1

Image, application 2

Image, application 3

Image, application 4

Image, application 5
