# Supplementary material for: Dataset for the performance of 15 lumbar movement control tests in nonspecific chronic low back pain
Source: Data Brief. 2022 Mar 16;42:108063. doi: 10.1016/j.dib.2022.108063 (PMC8965140; doi:10.1016/j.dib.2022.108063)
Supplement: Supplementary file 1 [file mmc1.docx]

**Incorrect** for all test movements 🡪 Lumbar spine moves too early and/ or too excessive. Patient is not aware of movement of the low back, cannot dissociate movement of the lumbar spine from movement of other body parts.

| Test No. | **Starting position** | **End position** | **Incorrect**  **(visually assessed)** |
| --- | --- | --- | --- |
| **1** | Test instruction:  Bend your upper body forward, try to reach the floor with your fingertips. Let your arms hang loosely and keep your knees straight | | Lumbar spine initiates movement and contributes more than hip and thoracic spine;  lumbar spine moves >25° ;  EOR excessive lumbar flexion;  Return ->lumbar spine unrolls late |
| Forward bend | **LMC in Flexion** | |  |
|  | **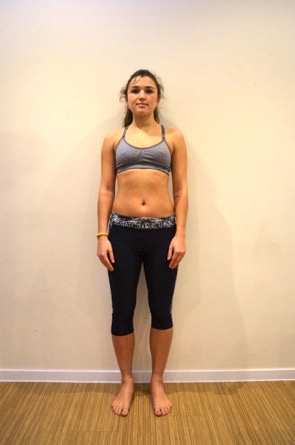** | **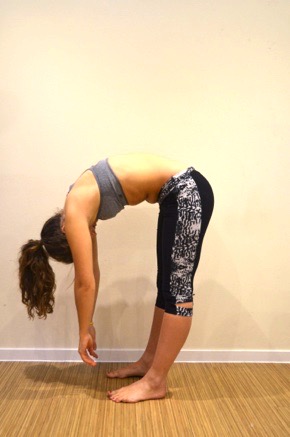** |  |
| **2** | Test instruction:  Lean your upper body back. Keep your arms crossed in front of your chest and keep your knees straight. | | Initiates with excessive forward pelvic sway (>10cm) or anterior pelvic tilt and lumbar extension;  Hip and thoracic spine moves later and contribute less  (hip >10-15°)  Return to neutral lumbar extension + anterior pelvic tilt persists and recovers later |
| Backward arching | **LMC in Extension** | |  |
|  | **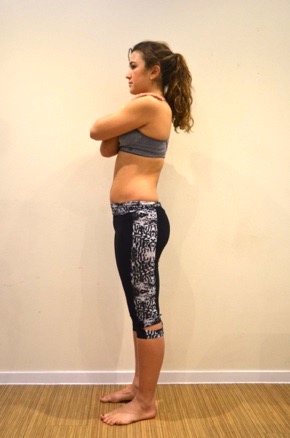** | **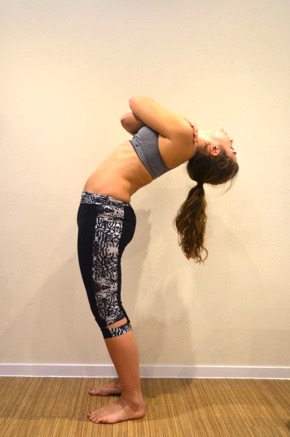** |  |
| **3** | Test instruction:  Slowly raise both arms upwards towards the ceiling. Stay upright and do not change the posture of your spine. | | No dissociation between movement of the lumbar spine from shoulder flexion;  increased lumbar spine ROM during mid-range shoulder flexion;  Pelvic drift forwards |
| Arm lift | **LMC in Ext or Flex** | |  |
|  | **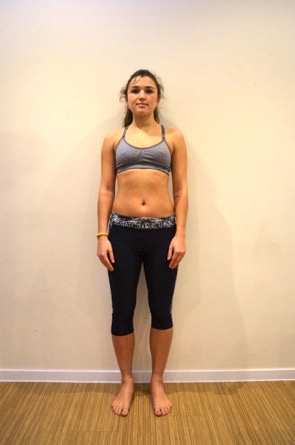** | **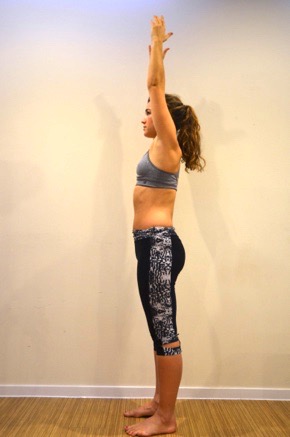** |  |

| **4** | Test instruction:  Lift your right / left leg as if you were going to climb a step. The upper body should remain upright. | | Uncontrolled and excessive movement of the lumbar spine in extension, flexion or rotation/lateral flexion, demonstrated by excessive movement of the pelvis, hip adduction or lateral trunk flexion. Lateral shift of the belly button > 10 cm (difference between sides >2cm) ; |
| --- | --- | --- | --- |
| One-leg stance | **LMC in Ext, Flex or Rot/Lat flex** | |  |
|  | **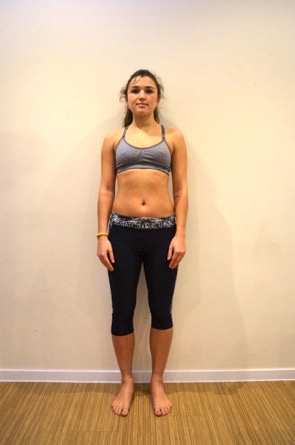** | **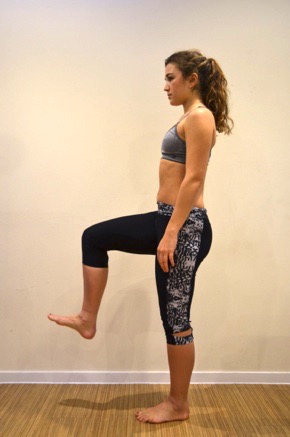** |  |
| **5** | Test instruction:  Straighten your right / left knee until your foot is approximately at the level of the knee joint. Only perform the movement as far as you can go while keeping the lumbar spine upright. | | Too early (often during beginning or mid range of knee extension) and too excessive lumbar flexion or rotation / lateral flexion during knee extension. Patient is not aware of movement of their lumbar spine  Note: Incorrect in one direction only |
| Sitting knee extension | **LMC in Flex or Rot/Lat flex** | |  |
|  | **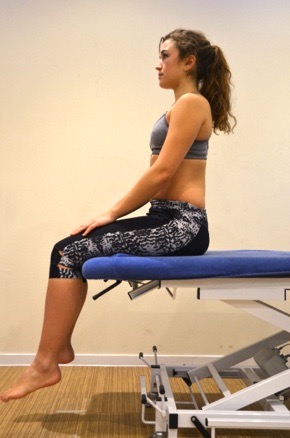** | **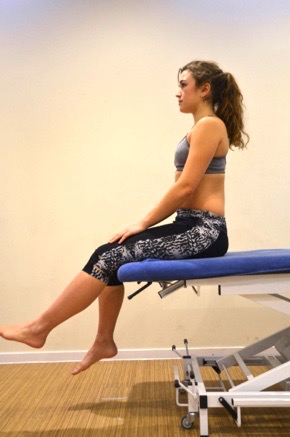** |  |
| **6** | Test instruction:  Sit upright on the bench with both hands on your thighs. Now bend your neck and lower your gaze towards the floor. Stop the movement as soon as the lumbar spine begins to move. | | Lumbar spine starts to flex before full thoracic spine flexion is achieved;  Difficulty to dissociate lumbar spine from independent thoracic spine flexion |
|  | **LMC in Flex** | |  |
| Chest drop | **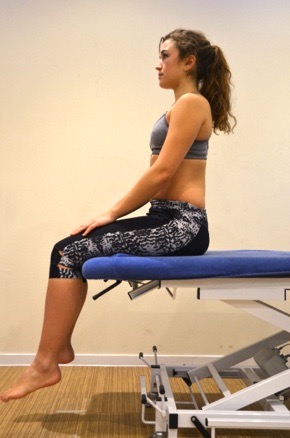** | **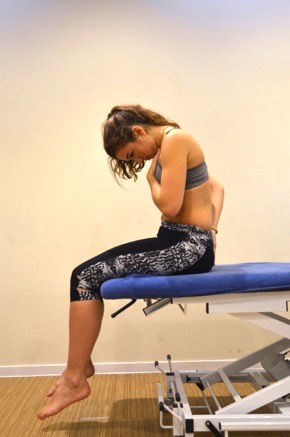** |  |
| **7** | Test instruction:  Slide your heel up the bed (hip flexion to approximately 45°). The heel should remain on the bed. The pelvis and lumbar spine should not move. | | Asymmetry of the ASIS as the hip flexes and returns;  No independent hip flexion possible;  Pelvic tilt with a consequence of lumbar spine extension;  Pelvic rotation with a consequence of lumbar spine rotation;  Pelvic straightening with a consequence of lumbar spine flexion |
| Single heel slide | **LMC in Ext, Flex, or Rot/Lat flex** | |  |
|  | **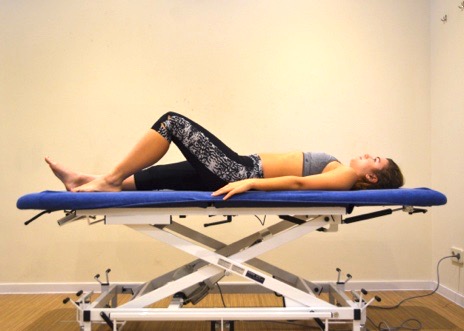** | **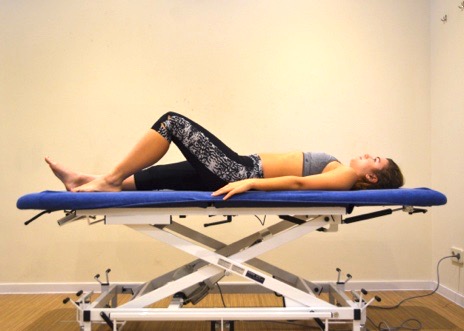** |  |
| **8** | Test instruction:  Start with knees bent, feet on the bench. Now lift both legs at the same time, keeping your knees bent. Stop when you reach 90 degrees hip flexion, or when instructed. Hold the position you have reached briefly. During the entire movement, you should keep the lumbar spine in the same starting position. The pressure on your back on the mat must not change. | | Lumbar spine starts to flex before achieving 90° of hip flexion;  Lumbar spine starts to extend before achieving 90° of hip flexion;  During return lumbar spine starts to extend before the feet reach the floor;  Bulging abdominal wall |
| (double) Leg lift & hold | **LMC in Ext or Flex** | |  |
|  | **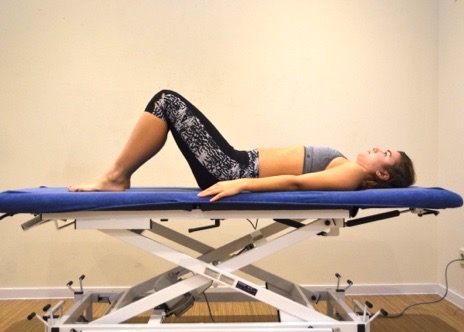** | **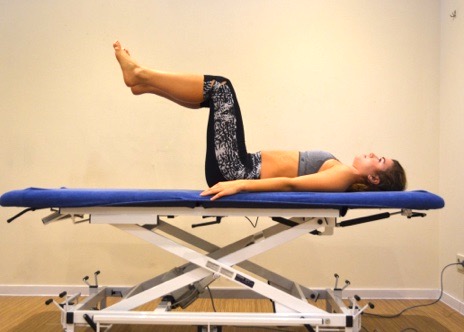** |  |
| **9** | Test instruction:  Start with knees bent, feet on the bench. Lower the right / left leg to the side. Both feet stay on the bench. When I say stop (45°), bring the leg back to the upright position. During the entire movement, you should keep the lumbar spine in the same starting position. | | Pelvis begins to rotate before 45° hip rotation;  Patient is unable to dissociate movement in the hip from lumbar spine and pelvis;  Positive test possible unilateral or bilateral |
| (single) Bend knee fall out | **LMC in Ext, Flex or Rot/Lat flex** | |  |
|  | **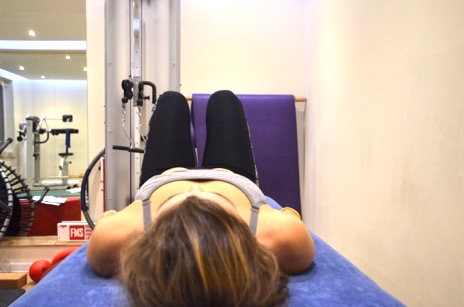** | **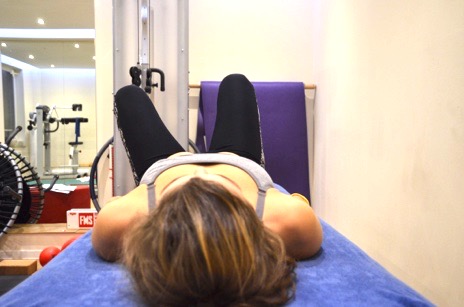** |  |
|  |  |  |  |
| **10** | Test instruction:  Bend your right / left knee until instructed to stop (120°). The thigh remains on the mat. During the entire movement, keep the lumbar spine in the same starting position. | | Lumbar spine extension before knees reach 120° flexion;  Lumbar spine rotation/lateral flexion before knees reach 120° flexion;  Pelvic anteriorly tilts or pelvic rotation before knee reaches 120° flexion  Positive test possible unilateral or bilateral |
| (single)  Prone knee flexion | **LMC in Ext or Rot/Lat flex** | |  |
|  | **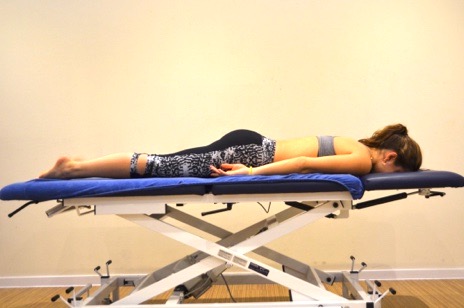** | **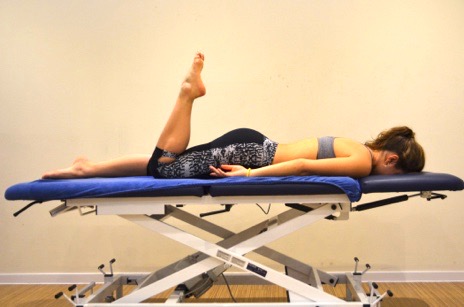** |  |
| **11** | Test instruction:  Lift your right / left knee slowly until instructed to stop (10° hip extension). The knee should remain straight. The lumbar spine must not move. | | Lumbar spine extension before the hip reaches 10° extension;  Lumbar spine rotation/lateral flexion before the hip reaches 10° extension;  Pelvic anteriorly tilts before hip reaches 10° extension  Positive test possible unilateral or bilateral |
| Single hip extension | **LMC in Ext or Rot/Lat flex** | |  |
|  | **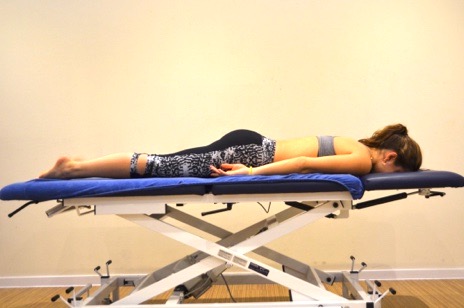** | **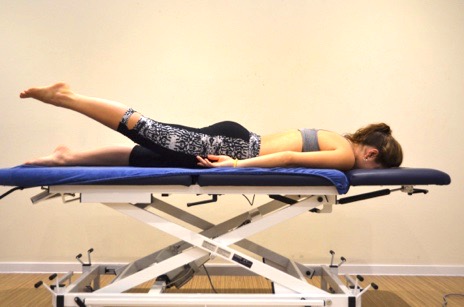** |  |
| **12** | Test instruction:  Bend your right / left knee and slowly lower your foot outwards and inwards. You should keep the pelvis in the same starting position. | | Lumbar spine rotation/ lateral flexion before hip reaches 30° medial and / or lateral rotation  Patient cannot dissociate between hip rotation and lumbopelvic rotation  Positive test possible unilateral or bilateral |
| Single hip rotation | **LMC in Rot./Lat flex** | |  |
|  | **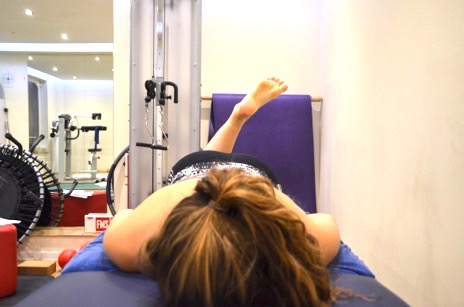** | **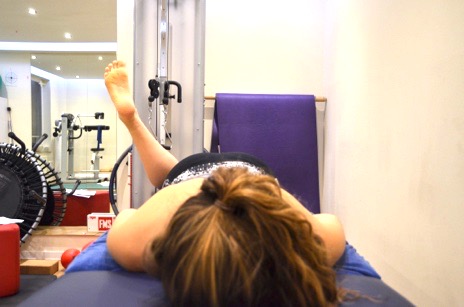** |  |
| **13** | Test instruction:  Rock your buttocks towards your feet (120° hip flexion). The back remains in the same starting position. | | - Flexion of lumbar spine during first 50% of motion (Lumbar spine flexion before hip reaches 120° flexion) - Patient is not aware of movement of their low back |
| Rocking backward | **LMC in Flex** | |  |
|  | **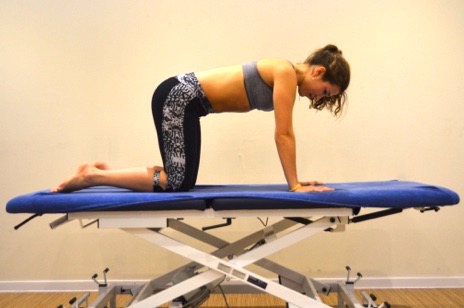** | **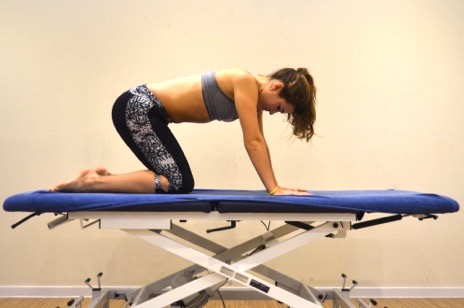** |  |
|  |  |  |  |
| **14** | Test instruction:  Rock forwards on your knees and shift your weight towards your hands (0° hip extension). The back remains in the same starting position. | | - Forward movement leads to marked lumbar spine extension usually seen during first 50% of motion. - Patient is not aware of movement of their low back |
| Rocking forward | **LMC in Ext** | |  |
|  | **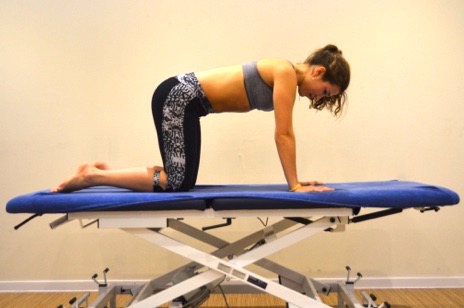** | **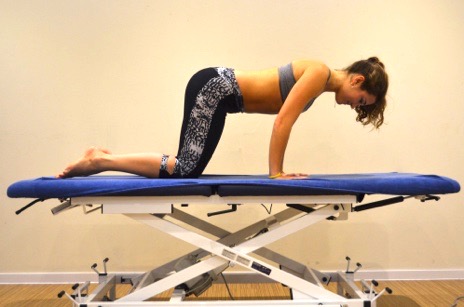** |  |
| **15** | Test instruction:  Lay on your side with your hips and knees bent, flexed to 45°. Both knees and both heels remain touching. Slowly raise your upper knee towards the ceiling until instructed to stop (15° hip abduction). The back remains in the same starting position. | | Lumbar spine extension before hip reaches 15° abduction  Lumbar spine flexion before hip reaches 15° abduction  Lumbar spine rotation/lateral flexion before hip reaches 15° abduction  Positive test possible unilateral or bilateral |
| Top leg turn | **LMC in Ext, Flex or Rot/Lat flex** | |  |
|  | **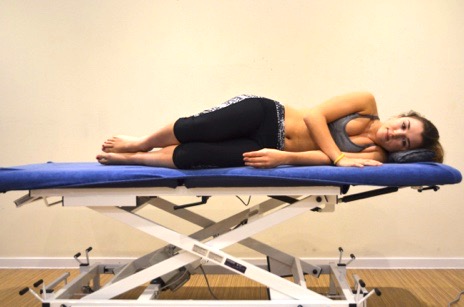** | **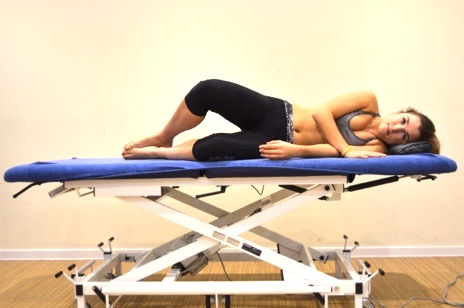** |  |
