# Supplementary material for: Dataset for the performance of 15 lumbar movement control tests in nonspecific chronic low back pain
Source: Data Brief. 2022 Mar 16;42:108063. doi: 10.1016/j.dib.2022.108063 (PMC8965140; doi:10.1016/j.dib.2022.108063)
Supplement: Supplementary file 2 [file mmc2.pdf]

APPENDIX 2

Test protocol

Proband ID:

Test No.	Initial Position	Test movement	Side (right / left)	Test result<br>1. Please mark maximum two crosses:<br>One cross for boxes 0, 1, 2, 3 or 5 and optional one cross for box 4<br>2. Please note tests performed both-sided<br>3. Please note the corresponding value in box Value after test session<br>e.g. incorrect in Ext (1) and existed pain (4) = 1(4); incorrect in Flex = 2						Value
				Correct (0)	Incorrect in Ext (1)	Incorrect in Flex (2)	Incorrect in Rot/Lat flex (3)	Pain (4)	Test not feasible (4)	
1	Standing	Forward bend		0	/	2	/	4	5	
2		Backward arching		0	1	/	/	4	5	
3		Arm lift		0	1	2	/	4	5	
4		One-leg stance	R	0	1	2	3	4	5	
			L	0	1	2	3	4	5	
5	Sitting	Sitting knee extension	R	0	/	2	3	4	5	
			L	0	/	2	3	4	5	
6		Chest drop		0	/	2	/	4	5	
7	Supine	Single heel slide	R	0	1	2	3	4	5	
			L	0	1	2	3	4	5	
8		Leg lift & hold		0	1	2	/	4	5	
9		Bend knee fall out	R	0	1	2	3	4	5	
			L	0	1	2	3	4	5	

APPENDIX 2

Test protocol

Proband ID:

**Test No.**	**Initial position**	**Test movement**	Side (right / left)	**Correct (0)**	**Incorrect in Ext (1)**	**Incorrect in Flex (2)**	**Incorrect in Rot/Lat flex (3)**	**Pain (4)**	**Test not feasible (5)**	Value
				**Test result**<br>1. Please mark maximum <u>two</u> crosses:<br><u>One</u> cross for boxes 0, 1, 2, 3 or 5 and optional <u>one</u> cross for box 4<br>2. Please note tests performed both-sided<br>3. Please note the corresponding value in box Value after test session<br>e.g. Incorrect in Ext (1) and existed pain (4) = 1(4); Incorrect in Flex = 2						
**10**	Prone	Prone knee flexion	R	0	1	/	3	4	5	
			L	0	1	/	3	4	5	
**11**		Single hip extension	R	0	1	/	3	4	5	
			L	0	1	/	3	4	5	
**12**		Single hip rotation	R	0	/	/	3	4	5	
			L	0	/	/	3	4	5	
**13**	4-point kneeling	Rocking backward		0	/	2	/	4	5	
**14**		Rocking forward		0	1	/	/	4	5	
**15**	Side-lying	Top leg turn	R	0	1	2	3	4	5	
			L	0	1	2	3	4	5	
